# Bacteriocins from the rhizosphere microbiome – from an agriculture perspective

**DOI:** 10.3389/fpls.2015.00909

**Published:** 2015-10-30

**Authors:** Sowmyalakshmi Subramanian, Donald L. Smith

**Affiliations:** Department of Plant Sciences, Macdonald Campus/McGill University, Sainte-Anne-de-BellevueQC, Canada

**Keywords:** *Bacillus* species, *Bacillus thuringiensis*, bacteriocins, anti-microbial peptides

## Abstract

Bacteria produce and excrete a versatile and dynamic suit of compounds to defend against microbial competitors and mediate local population dynamics. These include a wide range of broad-spectrum non-ribosomally synthesized antibiotics, lytic enzymes, metabolic by-products, proteinaceous exotoxins, and ribosomally produced antimicrobial peptides (bacteriocins). Most bacteria produce at least one bacteriocin. Bacteriocins are of interest in the food industry as natural preservatives and in the probiotics industry, leading to extensive studies on lactic acid bacteria (colicin produced by *Escherichia coli* is a model bacteriocin). Recent studies have projected use of bacteriocins in veterinary medicine and in agriculture, as biostimulants of plant growth and development and as biocontrol agents. For example, bacteriocins such as Cerein 8A, Bac-GM17, putidacin, Bac 14B, amylocyclicin have been studied for their mechanisms of anti-microbial activity. Bac IH7 promotes tomato and musk melon plant growth. Thuricin 17 (Th17) is the only bacteriocin studied extensively for plant growth promotion, including at the molecular level. Th17 functions as a bacterial signal compound, promoting plant growth in legumes and non-legumes. In *Arabidopsis thaliana* and *Glycine max* Th17 increased phytohormones IAA and SA at 24 h post treatment. At the proteome level Th17 treatment of 3-week-old *A. thaliana* rosettes led to >2-fold changes in activation of the carbon and energy metabolism pathway proteins, 24 h post treatment. At 250 mM NaCl stress, the control plants under osmotic-shock shut down most of carbon-metabolism and activated energy-metabolism and antioxidant pathways. Th17 treated plants, at 250 mM NaCl, retained meaningful levels of the light harvesting complex, photosystems I and II proteins and energy and antioxidant pathways were activated, so that rosettes could better withstand the salt stress. In *Glycine max*, Th17 helped seeds germinate in the presence of NaCl stress, and was most effective at 100 mM NaCl. The 48 h post germination proteome suggested efficient and speedier partitioning of storage proteins, activation of carbon, nitrogen and energy metabolisms in Th17 treated seeds both under optimal and 100 mM NaCl. This review focuses on the bacteriocins produced by plant-rhizosphere colonizers and plant-pathogenic bacteria, that might have uses in agriculture, veterinary, and human medicine.

## Introduction To Bacteriocins

Microbial population dynamics are primarily controlled by the products bacteria produce and excrete into their environs. This versatile and a dynamic suit of compounds order the defense mechanisms, rightly described as “a never ending arms race” (Riley, 1998) against microbial competitors and also as signaling compounds for plant colonization in a given soil. The excreted bacterial compounds we recognize now include a wide range of broad-spectrum non-ribosomally synthesized antibiotics, lytic enzymes (lysozymes), metabolic by-products such as organic acids, proteinaceous exotoxins, and chromosomally and/or ribosomally produced antimicrobial peptides, referred to as bacteriocins that are of particular importance in bacterial defense. It is supposed that most bacteria produce at least one bacteriocin.

Bacteriocins are extracellular substances produced by bacteria having distinctive morphological and biochemical characteristics, ranging from a very low to high molecular weight complexes, wherein the activity is predominantly associated with a protein. They are mostly synthesized from plasmids, but many are of chromosomal origin as well and are synthesized at various stages of bacterial growth and under various environmental conditions (Daw and Falkiner, 1996); they affect the growth of related bacterial species. Bacteriocins are grouped into four distinct classes based on peptide characteristics such as post translational modifications, side chains, heat stability, N-terminal sequence homology, and molecular weight (Klaenhammer, 1993). *Bacillus* species were first reported to produce bacteriocins in 1976 and the diversity of these is well described in the review by Abriouel et al. (2011). The low-molecular-weight bacteriocins of the Gram-positive bacteria were reported to demonstrate bactericidal activity, mainly against certain other Gram-positive bacteria (Tagg et al., 1976). The most studied bacteriocin is colicin of the Enterobacteriaceae (Pugsley, 1984). Due to their commercial importance as natural preservatives, and as therapeutic agents against pathogenic bacteria, these antimicrobial peptides have been a major area of scientific research (Tagg et al., 1976; Jack et al., 1995; de la Fuente-Salcido et al., 2013). Nisin, synthesized by *Lactococcus lactis*, is the only bacteriocin generally regarded as safe for human consumption (GRAS) but has limited usage since it is ineffective against Gram-negative bacteria (Olasupo et al., 2003) necessitating exploration of newer bacteriocins. Hence, this review is an update regarding the bacteriocins produced by plant-rhizosphere colonizers and those from plant-pathogenic bacteria that might have uses in agriculture and veterinary or human medicine.

## Bacteriocins From Rhizosphere Bacteria

The bacteriocin cerein7, from *Bacillus cereus* with a mass of 3.94 kDa was the first to be isolated from this species (Oscáriz et al., 1999) although other bacillus species such as *B. thuringiensis*, *B. subtilis*, *B. stearothermophilus*, *B. licheniformis*, *B. megaterium*, and *B. cereus* were reported earlier to produce bacteriocin like products, of which subtilin from *B. subtilis* has been studied widely. The earliest studies of bacteriocin mode of action focused on *Rhizobium lupini* isolated from root nodules of lupines that harbor two strains of the species 16-2 and 16-3, the latter of which produces a bacteriocin to inhibit the growth of its closely associated 16-2 strain (Lotz and Mayer, 1972). A comparison of these two strains revealed that bacteriocin activity of one bacteria can be neutralized by lipopolysaccharides of other associated bacteria, by micellar modulation of LPS for bacteriocin adsorption. This bacteriocin neutralizing activity, as seen in bacteriocin sensitive *R. lupini 16-2* (Pfister and Lodderstaedt, 1977), leads one to wonder why certain bacteria are able to adapt and colonize plant roots more effectively, despite their sensitivity to bacteriocins. Now we are aware that bacteriocin resistance can be innate or acquired and this varies across strains of the same bacterial species. In general changes to the bacterial cell wall resulting in loss of bacteriocin insertion or binding regions, sequestration of bacteriocins, export or degradation of bacteriocins have been adopted by Gram-positive bacteria (de Freire Bastos et al., 2015).

Cerein 8A isolated from *B. cereus* 8A, interferes with cell membrane integrity and causes cell wall damage (Bizani et al., 2005), which is seen to be the mode of action of many bacteriocins. (Please refer to **Table 1** for bacteriocins from *B. thuringiensis* and examples of other bacteria). Bacteriocin Bac-GM17 from the rhizosphere bacteria *Bacillus clausii* strain GM17 of *Ononis angustissima* Lam. is a 5.158 kDa monomer protein with a unique sequence and having bactericidal effect on *Agrobacterium tumefaciens* C58 and fungistatic effect on *Candida tropicalis* R2 CIP203 (Mouloud et al., 2013). The bacteriocin putidacin, produced by *Pseudomonas putida* strain BW11M1, isolated from banana root, is very similar to mannose-binding plant lectins (Parret et al., 2003). Amylocyclicin, a 6.381 kDa peptide from *Bacillus amyloliquefaciens* FZB42, is a novel circular, ribosomally synthesized bacteriocin with high antibacterial activity to closely related Gram-positive bacteria (Scholz et al., 2014). *B. subtilis* strain 14B produces Bac 14B, a 21 kDa bacteriocin that is effective against crown gall disease caused by *A. tumefaciens* (Hammami et al., 2009). *B. subtilis* strain IH7 produces a bacteriocin Bac IH7 which is reported to be a plant growth promotor. Tomato and muskmelon treated with Bac IH7 showed enhanced germination percentage and increased shoot weight and height and root lengths; it also served as a biocontrol for *Alternaria solani* and other seed borne pathogens (Hammami et al., 2011). A 25–35 kDa bacteriocin from *Lysinibacillus* jx416856, a bacteria isolated from fruit and vegetable waste, was observed to inhibit food borne pathogens such as *Staphylococcus aureus*, *Staphylococcus epidermidis*, and *B. cereus* (Ahmad et al., 2014).

**Table 1:** Bacteriocins identified from *Bacillus thuringiensis* and examples of other bacteriocin producers.

*Bacillus thuringiensis* strain	Name of Bacteriocin identified	Molecular weight	Reference
HD-2	Thuricin	>950 Da	Favret and Yousten, 1989
ssp. *tochigiensis* HD868	Tochicin	10.5 kDa	Paik et al., 1997
BMG1.7	Thuricin 7	11.6 kDa	Cherif et al., 2001
B439	Thuricin 439	3 kDa	Ahern et al., 2003
ssp. *entomocidus* HD9	Entomocin 9	12.4 kDa	Cherif et al., 2003
BUPM4	Bacthuricin F4	3.1 kDa	Kamoun et al., 2005
NEB17	Thuricin 17	3.16 kDa	Gray et al., 2006b
ssp. *entomocidus* HD110	Entomocin 110	4.8 kDa	Cherif et al., 2008
ssp. *entomocidus* HD198	Thuricin S	3.1 kDa	Chehimi et al., 2007
ssp. *morrisoni* (LBIT 269	Morricin 269	10 kDa	Barboza-Corona et al., 2007; de la Fuente-Salcido et al., 2008
ssp. *kurstaki* (LBIT 287)	Kurstacin 287	10 kDa	Barboza-Corona et al., 2007; de la Fuente-Salcido et al., 2008
ssp. *kenyae* (LBIT 404)	Kenyacin 404	10 kDa	Barboza-Corona et al., 2007; de la Fuente-Salcido et al., 2008
ssp. *entomocidus* (LBIT 420)	Entomocin 420	10 kDa	Barboza-Corona et al., 2007; de la Fuente-Salcido et al., 2008
ssp. *tolworthi* (LBIT 524)	Tolworthcin 524	10 kDa	Barboza-Corona et al., 2007; de la Fuente-Salcido et al., 2008
SF361 [isolated from honey]	Thuricin H	3.1 kDa	Lee et al., 2009a
DPC6431	Thuricin CD	2.763/2.861 kDa	Rea et al., 2010
BUPM103	Bacthuricin F103	11 kDa	Kamoun et al., 2011
ssp. *kurstaki* Bn1 [isolated from a Hazel nut pest]	Bn1	3.193 kDa	Ugras et al., 2013
**Examples from *Bacillus* and other bacterial species**			
*Bacillus cereus*	Cerein 7	3.94 kDa	Oscáriz et al., 1999
*Pseudomonas putida* BW11M1	Putidacin		Parret et al., 2003
*Bacillus cereus* 8A	Cerein 8A		Bizani et al., 2005
*Clavibacter michiganensis* ssp. *michiganensis*	Michiganin A		Holtsmark et al., 2006
*Bacillus licheniformis*	BL8	1.4 kDa	Smitha and Bhat, 2012
*Bacillus clausii*	Bac GM17	5.158 kDa	Mouloud et al., 2013


Some plant pathogens have also been found to produce bacteriocins. Phytopathogenic strain *Erwinia carotovora* NA4, isolated from diseased fruits and vegetables, produces the bacteriocin erwiniocin NA4 and *Agrobacterium radiobacter* NA5 (pepper rhizosphere isolate) produces agrocin NA5 (Jabeen et al., 2004). Tomato pathogen *Clavibacter michiganensis* ssp. *michiganensis* produces a bacteriocin michiganin A, which inhibits the growth of another pathogen *C. michiganensis* subsp. *sepedonicus*, which causes ring rot of potatoes. This bacteriocin also has similarity to a type B lantibiotic produced by the actinomycete *Actinoplanes liguriae* (Holtsmark et al., 2006). *Pseudomonas syringae* pv. *syringae* produces S-type pyocins (Feil et al., 2005), which are also produced by the opportunistic human pathogen *Pseudomonas aeruginosa*. Carocin S1, is a 55 kDa bacteriocin from *Pectobacterium carotovorum* (previously known as *E. carotovora* ssp. *carotovora*; Holtsmark et al., 2008).

*Bacillus thuringiensis* is the most studied *Bacillus* species due to its interesting array of excreted proteins. *B. thuringiensis* is a Gram-positive spore-forming bacterium characterized and distinguished from closely related *Bacillus* species by its ability to synthesize characteristic endotoxins that are active against diptera, coleoptera, and Lepidoptera larvae (Schnepf et al., 1998; Palma et al., 2014). Widely used as a bioinsecticide, it also accounts for about 90% of the commercially available biopesticides produced (Chattopadhyay et al., 2004), apart from the Bt genes incorporation in several commercial crops, the proteomics and genomics of which is well known (Ibrahim et al., 2010; de la Fuente-Salcido et al., 2013). *B. thuringiensis* was discovered as early as 1901 in Japan by bacteriologist S. Ishiwata as an isolate from diseased *Bombyx mori* (L.) larvae and was named Sottokin meaning “Sudden death bacillus”. A similar study by Ernst Berliner, described *B. thuringiensis* as the causal organism of insect death isolated from *Anagasta kuehniella* (Zeller) larvae from Thuringia, Germany in 1915 [Beegle and Yamamoto, 1992 (a very good review for the history of *B. thuringiensis*)].

*Bacillus thuringiensis* strains have been found to produce bacteriocins such as thuricin (>950 kDa; Favret and Yousten, 1989), tochicin (10.5 kDa; Paik et al., 1997), thuricin 7 (11.6 kDa; Cherif et al., 2001), thuricin 439A and thuricin 439B (2.9 and 2.8 kDa, respectively; Ahern et al., 2003), entomocin 9 (Cherif et al., 2003), bacthuricin F4 (3.160 kDa; Kamoun et al., 2005), thuricin 17 (3.162 kDa; Gray et al., 2006b), etc. A comprehensive list of known bacteriocins is provided in **Table 1**. New bacteriocins are being discovered regularly. Raddadi et al., (2009) evaluated 16 strains of *B. thuringiensis* for their capability to protect plants from phytopathogens. Among them, Bt HD868 tochigiensis and Bt HD9 entomocidus strains were observed to be the least cytotoxic, and hence potentially acceptable for the food industry and field crop application for protection against deleterious bacteria. This compatibility was based on the levels of autolysins, bacteriocins and AHL-lactonases, and antibiotic Zwittermicin A activities. Further these strains were also active against fungal diseases caused by *Aspergillus niger*, *Aspergillus fumigatus*, *Aspergillus flavus*, *Cryphonectria parasitica*, *Fusarium oxysporum*, *Monilia sitophila*, *Monilia hiemalis*, *Penicillium digitatum*, and *Rhizopus* sp. (Raddadi et al., 2009). While many *B. thuringiensis* strains have been identified and bacteriocins were isolated and characterized to an extent, none of these bacteriocins have been studied for plant growth promotion as extensively as thuricin 17 from *B. thuringiensis* NEB17.

*Bacillus thuringiensis* NEB17 was isolated from soybean root nodules as putative endophytic bacteria in 1998 in Prof. Donald Smith’s laboratory at McGill University. When co-inoculated with *Bradyrhizobium japonicum* under nitrogen free conditions, this bacterium promoted soybean growth, nodulation, and grain yield (Bai et al., 2002b, 2003). Subsequently, the causative agent of plant growth promotion, a bacteriocin, was isolated from *B. thuringiensis* NEB17, and is now referred to as thuricin 17 (Gray et al., 2006b). Initially, its partial sequence was determined (Gray et al., 2006a), and its full sequence has since reported (Lee et al., 2009b). Thuricin 17 is a low molecular weight peptide of 3.162 kDa, stable across a pH range of 1.0–9.25, highly heat resistant and is inactivated by treatment with proteolytic enzymes. Based on its N-terminal sequence homology of Th17 and that of bacthuricin F4, a new class of bacteriocins, class IId was proposed (Gray et al., 2006b). The bacteriocins produced by *B. thuringiensis* strain NEB17 (Th17) and *B. thuringiensis* ssp. *kurstaki* BUPM4 (bacthuricin F4 – 3160.05 Da) have been reported to show functional similarities and anti-microbial activities (Jung et al., 2008a).

In addition, Th17, applied as leaf spray and root drench, has positive effects on soybean and corn and stimulated plant growth. The leaves of 2-week-old soybean leaves sprayed with Th17 showed increased activities of lignification-related and antioxidative enzymes and their isoforms (Jung et al., 2008a; Lee et al., 2009b); this constituted the first report of plant growth stimulation by a bacteriocin. Recent research on Th17 has highlighted its plant growth promotion and abiotic stress alleviation properties. It was found that at 24 h after exposure to Th17, *Arabidopsis thaliana* Col-0 rosettes showed decreased levels of cytokinins, gibberellins, JA, and ABA; and an increase in IAA (85.39%) and SA (42.21%) as compared to untreated control plants. *A. thaliana* responded positively to treatment with Th17 in the presence of salt stress (up to 250 mM NaCl). Shotgun proteomics of unstressed and 250 mM NaCl stressed *A. thaliana* rosettes (7 days post stress) in combination with Th17 revealed carbon and energy metabolic pathways being affected under both unstressed and salt stressed conditions. Chloroplast proteins and proteins of photosystem I and II that are generally strongly and negatively affected by salt stress and PEP carboxylase, Rubisco-oxygenase large subunit, and pyruvate kinase, were some of the noteworthy proteins enhanced (>2-fold changes in the activation of the carbon and energy metabolism pathway) by Th17 application, along with other stress related proteins. These findings suggest that the proteome of *A. thaliana* rosettes is altered by the bacterial signal, and more so under salt stress, thereby imparting a positive effect on plant growth under high salt stress (Subramanian, 2014).

Application of Th17, under water stress conditions, to 1 month-old soybean plants increased plant biomass by 17%, root biomass by 37% and root nodule biomass by 55%, and also the amount of abscisic acid in soybean roots by 30% (Prudent et al., 2014). Application of Th17 to soybean seeds (variety Absolute RR) caused accelerated seed germination under salt stress of up to 150 mM NaCl, with the best response seen at 100 mM NaCl. Shotgun proteomics of unstressed and 100 mM NaCl stressed seeds (48 h) in combination with Th17 revealed that carbon, nitrogen and energy metabolic pathways were affected under both unstressed and salt stressed conditions. PEP carboxylase, Rubisco oxygenase large subunit, pyruvate kinase, alcohol dehydrogenase, and isocitrate lyase were some of the noteworthy proteins enhanced (>2-fold changes), by the signal, along with antioxidant glutathione-*S*-transferase and other stress related proteins. The up-regulation of PEP carboxylase and a marked down-regulation of α- and β-subunits of conglycinin, glycinin, as compared to the control treatment, is indicative of efficient storage protein utilization in conjunction with thioredoxin. These findings suggest that the germinating seeds alter their proteome based on bacterial signals and on stress level; the specificity of this response plays a crucial role in organ maturation and transition from one stage to another in a plant’s life cycle; understanding this response is of fundamental importance in agriculture and, as a result, global food security (Subramanian, 2014). As observed in our experiments, the effective concentration of bacteriocin for enhanced plant growth and production is in the order of nanomolar (10^-9^ M), which makes it economically viable as method to decrease the use of energy based fertilizers and chemicals used in agricultural crop production systems.

## Bacteriocins In The Veterinary Industry And Human Medicine

Bacteriocins from *B. thuringiensis* also have proven to be of importance in veterinary medicine. *S. aureus* causes clinical and subclinical bovine mastitis, which is difficult to treat due to increased frequency of resistance to antimicrobial agents. *S. aureus* isolates recovered from milk composite samples of Holstein lactating cows in Mexico were evaluated for susceptibility of the isolates to 12 antibiotics and five bacteriocins from *B. thuringiensis*. *S. aureus* isolates were resistant to penicillin (92%), dicloxacillin (86%), ampicillin (74%), and erythromycin (74%) and susceptible to gentamicin (92%), trimethoprim (88%) and tetracycline (72%). *S. aureus* isolates also showed susceptibility to the five bacteriocins synthesized by *B. thuringiensis*, morricin 269 and kurstacin 287 followed by kenyacin 404, entomocin 420 and tolworthcin 524 suggesting an alternate method of controlling bovine mastitis (Barboza-Corona et al., 2009).

Mexican strains of *B. thuringiensis*, *B. thuringiensis* ssp. *morrisoni* (LBIT 269), *B. thuringiensis* ssp. *kurstaki* (LBIT 287), *B. thuringiensis* ssp. *kenyae* (LBIT 404), *B. thuringiensis* ssp. *entomocidus* (LBIT 420) and *B. thuringiensis* ssp. *tolworthi* (LBIT 524) produce proteinaceous Bt-BLIS bactericidal activities against *B. cereus* and *Vibrio cholera* (Barboza-Corona et al., 2007) but had no effect against Gram-negative bacteria such as *Escherichia coli*, *Shigella* species, and *P. aeruginosa*, all of which are human pathogens. Entomocin 9 was found to be bactericidal to *Listeria monocytogenes*, pathogenic *P. aeruginosa* and several fungi causing cell lysis of growing cells and non-toxic to Vero cells (Cherif et al., 2003). Thuricin 439, however, is a narrow spectrum bacteriocin capable of affecting growth of *B. cereus* (Ahern et al., 2003), while thuricin S is anti-*Listeria* (Chehimi et al., 2007) and a pore-forming bacteriocin (Chehimi et al., 2010). *B. cereus* cells protect themselves from Enterocin AS-48 produced by *Enterococcus faecalis* S48 by up-regulating the BC4207 membrane protein for probable membrane structure modulation (Burgos et al., 2009). While pyocins produced by *P. aeruginosa* strains have proven to be toxic by degrading DNA in sensitive bacterial cells (Parret and De Mot, 2002). *B. subtilis* strain LFB112 from Chinese herbs produces a 6.3 kDa bacteriocin that is effective against *E. coli*, *Salmonella pullorum*, *P. aeruginosa*, *Pasteurella multocida*, *Clostridium perfringens*, *Micrococcus luteus*, *Streptococcus bovis*, and *S. aureus* IVDC C56005, all of which are common domestic animal related pathogens (Xie et al., 2009).

Optimizing medium composition, incubation and agitation speed can result in enhancement of the production of some bacteriocins. For example, *B. thuringiensis* subsp. *kurstaki* strain, producing the bacteriocin Bacthuricin F4 when grown in TSB medium with an optimal carbon–nitrogen ratio of 9 increases the bacteriocin production fourfold (Kamoun et al., 2009). Bacteriocin *Bacillus* sp. YAS 1 could be increased 1.6-fold by this method. This bacteriocin had a wide pH range (1–13) as well as temperature (45–80°C) with antimicrobial activity to human pathogens such as *Clostridium*, *Staphylococcus*, *Enterococcus*, and *Salmonella*, and plant pathogens such as *E. amylovora*, and showing no effect on lactic acid bacteria (Embaby et al., 2014).

## Bacteriocins From Other Interesting Bacterial Sources

While most bacteriocins we know today have been isolated from the rhizosphere bacteria, bacteria producing bacteriocins are wide spread. A *B. subtilis* strain isolated from a Chinese fermented seasoning produces a 3.4 kDa bacteriocin that is active against *B. cereus* and *L. monocytogenes* (Zheng and Slavik, 1999). Maari, an alkaline fermented food condiment made from baobab tree seeds, is comprised of several strains of *B. subtilis*, all of which are necessary for enhancing the flavor and texture of the product. Three *B. subtilis* strains (B3, B122, and B222) isolated from maari produced bacteriocins that had antibacterial activities against *B. cereus* NVH391-98, a common opportunist human pathogen that contaminates maari (Kaboré et al., 2013). People in Burkina Faso and neighboring countries consume a fermented product called bilakga, derived from the seeds of *Hibiscus sabdariffa*. The fermentation concoction is largely comprised of *B. subtilis* subsp. *subtilis* and *Bacillus licheniformis* isolates. PCR detection of genes coding for surfactins and plipastatins (fengycins) suggested the production of subtilosin, subtilin and lipopeptide, while a protein (a probable bacteriocin) with a mass of 3.347 kDa was also isolated (Compaoré et al., 2013). *B. thuringiensis* ssp. *kurstaki* Bn1 isolated from hazel nut pest *Balaninus nucum* L. produces a bacteriocin Bt-Bn1, the first of its kind of insect origin. Like its potential commercial counterparts, this bacteriocin has antibacterial activity against *B. cereus*, *P. syringae*, and *P. lemoignei*, and other *B. thuringiensis* strains (Ugras et al., 2013).

## Conclusion

Excessive use of fertilizers and other chemicals in agriculture and multi-drug resistant microbes are two major challenges for scientists worldwide. Bacteriocins evaluated as plant growth promotors and those with disease suppression mechanisms are a viable option for efficient use in agriculture, to reduce the use of fertilizers and chemical inputs such as fungicides and insecticides. With respect to multi-drug resistance, bacteriocins can be interesting in commercial utilization as target proteins for replacing ineffective antibiotics or for combinatorial drug therapy both in veterinary and human medicine, apart from their use in food preservation.

## Conflict of Interest Statement

The authors declare that the research was conducted in the absence of any commercial or financial relationships that could be construed as a potential conflict of interest.
